# Surgery

**Published:** 1837-04

**Authors:** 


### SURGERY.

*On the Nature and Treatment of Lupus.* By Alexander Ure, M.D.

This is a valuable practical paper, of which we can only give a brief notice.—Dr. Ure confines the term Lupus to two varieties of erosive ulcer; one of which he terms *Erosive ulcer of the derma*, and the other *Erosive ulcer of the follicles*. The first is regarded as symptomatic, being generally dependent on disordered assimilation and nutrition: it is extremely obstinate, resisting all local applications, unless the morbid state of the general system is removed. Powerful escharotics, which are so effective in the other species, effect here merely a temporary suspension of the disease. The second form is more decidedly a local affection, and is readily curable by proper local applications. This variety frequently originates like a catarrhal affection of the Schneiderian membrane, which terminates in erosive ulceration, and eventually leads to perforation of the cartilaginous septum. After a time, the external surface of the nose becomes inflamed, and ulcerates; the ulceration having its seat principally in the sebaceous follicles, and all along preserving the tuberculous character. The dreadful havoc committed by this disease is well known. Dr. Ure says that this form of ulceration is often speedily checked, and the disease permanently cured, by the local use of the chloride of zinc, or a solution of the nitrate of zinc in strong nitric acid. The former is the remedy chiefly used, and is applied in the form of a paste, made with one part of the chloride and two or three parts of the anhydrous sulphate of lime; a modification of Canquoin's formula, originally proposed and introduced into practice by Dr. Ure. (See Medical Gazette, Dec. 19, 1835.) One or two applications of the paste will at most suffice to produce a proper eschar; and, when this is detached, the sore, now healthy, is to be treated with water dressing; or, if the surface be very uneven, with narrow strips of adhesive plaster, which are allowed to remain on till they drop off. Three cases illustrative of the successful treatment of this form of lupus are given:

1. The disease was of some years' standing, began in the mucous membrane, and progressively spread to the ala. There was tubercular ulceration of the follicles, and the septum was perforated by a large aperture. Many remedies had been tried, but in vain. A thin layer of the gypsum paste was laid over the sore, and, after the removal of the eschar, this healed in fifteen days.

2. Disease of seven years' standing. The ulceration had continued five years, and had destroyed the greater portion of the septum. There was an erosive tubercular ulcer of the left ala, which was in part eaten away. Numberless remedies, and, among others, iodine and sarsaparilla, had been tried in vain. The chloride-of-zinc paste produced a permanent cicatrix.

3. The disease of two years' standing. Corroding ulcer, of a marked tuberculous character, on the nose: it consisted of a group of irritable-looking tubercles, covered with crusts, with a dark-red hue of the adjacent skin. The cartilaginous septum was perforated, and a portion of the ala destroyed. A thin layer of the gypsum paste was applied, and, in a few weeks after, a healthy cicatrix occupied the site of the ulcer.—In each of these cases, the loss of substance is said to be inconsiderable.

We think the profession much indebted to Dr. Ure for this improvement in the treatment of so unmanageable a disease. *Med. Gazette, Dec.* 3, 1836.

---

*New Mode of Operating for Nævus.* By Mr. LISTON.

A CHILD, aged twenty months, was admitted into the North London Hospital, on the 2d of November, with a nævus, of the size of a pigeon's egg, on the face, near the nose. It had gradually increased to its present size, was compressible, and filled speedily on the removal of pressure. On the 7th, Mr. Liston made a crucial incision through the integuments covering the tumour, and carefully dissected back the four flaps, so as completely to expose the tumour. A needle, armed with a double ligature, was then passed through the base of the tumour, and another, in like manner, at right angles to the former. The needles being withdrawn, four ligatures remained, and were successively tied over the tumour, so as to comprehend the whole. Water dressings, at first cold, and afterwards warm, were applied. The case went on favorably, and the report on the 21st (the fourteenth day after the operation,) is, "The whole tumour is now come away, the swelling is abating, and the edges of the integument coming gradually together."

*Lancet, Dec.* 31, 1836.

---

*Cure of an Open Cancer of the Breast.* By ANDREW BUCHANAN, M.D. Glasgow.

THIS case is so far interesting as affording a proof that a genuine cancer of the mamma admits of being cured, although it supplies no new therapeutic means, nor adds to our confidence in the old: nature seems to be entitled to all the merit of the cure. The opinion of the experienced narrator of the case is quite sufficient to establish the cancerous nature of the disease, if this were not evident from the description given of it. The patient was forty years of age, and had laboured under the disease three years, when admitted into the Glasgow Infirmary, on the 21st January; but ulceration had only taken place a few weeks previously. She was dismissed in the end of March, with increase of the local disease; and this increased for some time afterwards, until the ulcer was "as broad as the mouth of a teacup, and very deep," with an excessively fetid discharge. Some time after this, she was advised to dress it with the common Unguent. Citrin. dil. of the Edinburgh Pharmacopœia; and under this treatment it gradually healed up. When seen in November by Dr. Buchanan, the cicatrix left by the sore was about two inches in diameter, indurated, and quite flat. The surrounding parts seemed quite healthy, but several of the axillary glands were enlarged. The general health was good. The only internal remedy taken by this patient was hydriodic acid (during the early period of the ulceration,) for forty-two days, the total quantity of iodine taken being 2460 grains. *Med. Gazette, Dec.* 17, 1836.

---

*Gangrene of the Leg from Disease of the Heart.* By W. CLARK, Esq., Surgeon, Devizes.

THIS is an interesting and curious case, the outlines of which we can only give: A woman, æt. 50, the subject of valvular disease of the heart with dilated auricles, got out of bed at night in a profuse perspiration, went down stairs, and, in opening the door of her house, placed her right heel on the damp brick floor. On the following morning, she was seized with severe pain in the right foot, darting upwards to the thigh, which was soon followed by loss of sensation and coldness. Next day, the limb was of a leaden hue, still cold and senseless, and no pulsation could be felt in the popliteal or posterior tibial arteries; but slight action in the femoral, under Poupart's ligament. Vesications appeared on the third day; a line of demarcation on the sixth; but the gangrene proceeded unchecked, and the woman died on the fifteenth day. A singular circumstance was observed during the progress of the mortification,—viz. the repeated change in colour of the limb, from the usual leaden, livid hue, to a bright scarlet, without any increase of temperature. On dissection, besides the primary disease of the heart, there was found a somewhat peculiar condition of the vessels of the lower extremities. The common iliac artery on both sides was small, not larger than the axillary; and the femoral not larger than an ordinary brachial. The femoral artery of the diseased limb was found gradually tapered downwards, until its cavity became obliterated above the part where the sloughing commenced; the coats being perfectly healthy. Both the iliac and femoral veins were unusually large; at least double their natural caliber. Mr. Clark thinks the smallness of the arteries may be accounted an auxiliary predisposing cause of the gangrene. *Med. Gazette, Feb.* 11, 1837.

---

*On the Treatment of Erysipelas by Bandaging.* By James Allan, Esq.

In this short communication the author advances nothing new; but we think the profession much indebted to him for recalling attention to a practice which we regard as of much practical importance, and which is much less practised than it ought to be. We are convinced that the principle of *pressure* to inflamed parts is capable of more extensive application than it has hitherto received. A striking instance of its value in a disease to which it had not been previously applied, was recently communicated in this Journal, (see Dr. Fricke, on the Treatment of Orchitis, vol. ii. p 253;) and we observe in recent periodical works several practical testimonies in its favour. Mr. Allan relates three cases,—two of erysipelas in the lower extremities, and one in the face; in all of which the application of the bandage was of speedy and permanent benefit; the pain caused by it being of very short duration. He also informs us that he has found bandages of very great service in removing the pain and swelling of joints which have been affected with acute rheumatism, after the reduction of the acuter degree of inflammation.

*British Annals of Medicine, Jan.* 27, 1837.

---

*Surgical Report of Cases treated in the Glasgow Royal Infirmary, from the* 1*st May,* 1835, *to the* 1*st August,* 1836. By John Macfarlane, m.d., Senior Surgeon.

This very valuable Report, which may serve as a model for similar documents, extends to upwards of fifty pages, and contains numerous interesting cases, with observations on the following subjects: 1. Dislocations; 2. Fractures; 3. Injuries of the head; 4. Abscess of the tibia; 5. Carcinoma; 6. Medullary sarcoma; 7. Fungus of the antrum; 8. Epulis; 9. Excision of the elbow-joint; 10. Aneurism; 11. Hernia; 12. Lithotomy; 13. Lumbar abscess; 14. Polypus of the rectum; 15. Secondary hemorrhage; 16. Traumatic phlebitis, and purulent deposits. We regret much that we cannot transfer to our pages more of the interesting practical matter with which this paper abounds: we must content ourselves with a brief extract from one or two of the sections.

*Carcinoma.* "Dr. Macfarlane justly laments the want of more extensive and accurate investigations respecting the results of operations for this disease, as it is only by this means that we can appreciate the chances of success from surgical interference. He is of opinion that, when this disease affects the mamma, whether involving the whole gland or in the form of tubercle, it is rarely that an operation proves ultimately successful. He says that, for some years past, he has made enquiries on the subject regarding his own cases or those of others; and the result has been very unsatisfactory. He has ascertained the termination of upwards of thirty cases, fourteen of which were operated on by himself; and in all, with one exception, the disease returned in a period varying from a few months to about three years. Some of these were exceedingly favorable, the disease being recent, and the adjoining lymphatic glands unaffected; yet it returned in almost every case, either in the axilla, near the old cicatrix, in some internal organ, or in one of the long bones; most frequently the former. We apprehend the experience of most surgeons, who are careful not to confound carcinoma with other diseases, will corroborate the results obtained by Dr. Macfarlane.

*Epulis.* "There are two varieties of this disease met with in practice; one simple or benign, and the other malignant. The former is small and defined, being seldom larger than a walnut, of a firm texture, and more or less pedunculated. It begins in the gum, frequently after abscess of the part, and but rarely affects the bone; and, as it increases, which it does very slowly, from being covered with a smooth membrane, it becomes granular on the surface. Its base is seldom large, even where the disease has been of long duration; and the tumour occasionally bleeds a little, but is attended with little or no pain. On its being removed by the knife or ligature, it may be repeatedly reproduced, unless the surface from which it springs be destroyed by the cautery or caustic; and, in general, the secondary and subsequent growths are more rapid in their formation, and of a softer texture, than the original tumour. The second variety is evidently malignant: it begins in the gum, at the root of one of the teeth, in the form of a small, ill-defined, spongy, and purple-coloured tubercle; it extends rapidly along the soft parts, which become swollen, vascular, and lobulated, loosening the teeth, dipping into the alveolar processes, involving the bone and palate, and often giving rise to profuse fetid discharge and to hemorrhage. It may extend over the entire jaw-bone, and among the bones of the face, so as to impede mastication and deglutition, contaminate the absorbent glands, and produce distressing deformity. In some cases the tumour possesses a sarcomatous hardness, and remains so throughout its progress; and in others it is soft and spongy; but in all, as it increases, it becomes more irregular on the surface, more fungoid in appearance, and firmer in texture.

*Excision of the Elbow-joint.* "The prejudice so long entertained against this operation is now yielding to the daily increasing number of cases in which it has proved successful, not only in removing the disease, but in preserving useful limbs. Although not a difficult, it is certainly a tedious and painful operation. The wound is extensive, and, from the disease of the soft parts generally present, profuse suppuration sometimes ensues. The cure is therefore protracted, and months must elapse before the functions of the arm are restored. In very unfavorable states of the system, some of these objections may perhaps warrant us in preferring amputation; but, in cases at all favorable, when the disease is confined to the articulation, and the system is able to bear the shock of the operation, and, it may be, the subsequent profuse discharges, we are not only warranted, but, I think, imperatively called upon, on account of the remote advantages it holds out, to have recourse to excision.

"In children, after excision, the parts sooner accommodate themselves to the loss which has been sustained, and the limb thus preserved sooner regains its strength and usefulness, than in adults. But, even in the latter class of cases, the ultimate success of the operation is also undoubted."

*Edinburgh Journal, Jan.* 1837.

---

*On the Treatment of Hydrocele by Acupuncture.* By D. LEWIS.

IN the Lancet of May 7th, 1836, Mr. Lewis communicated the interesting and important fact, that hydrocele might be cured by a single puncture with a fine needle. He says "a drop of fluid oozes out on withdrawing the needle, and in three days the swelling will completely disappear, no matter in what quantity the fluid may have been collected." It appears that the effect of the operation is not to evacuate the fluid through the puncture, but to cause its absorption. He informs us that, out of upwards of fifty cases, there has not been a single instance of failure, nor any consecutive inflammation. It appears that this mode of treatment is applicable to other cases of circumscribed dropsy; and Mr. Lewis informs us, that Dr. Thomas Davies finds it successful in removing fluids from the chest. Mr. L. says that the needle used in this operation "cannot be too fine, provided it be strong enough to penetrate through the integuments; for, the smaller the puncture, the less pain and inflammation ensue." *Lancet, January* 14, 1837.

---

### *Account of a Method of Operating for Hydrocele.*
### By Benjamin Travers, Esq.

This is essentially the same operation described in the preceding extract, but somewhat modified. The following is Mr. Travers's own account of his mode of proceeding, and the result of his experience of the practice.

"In the spring of 1836 I commenced the practice of the operation; first, making a single puncture with a trochar; secondly, with a fine sharp-pointed probe; thirdly, with an acupuncture needle of the largest size; and then planting several punctures at equal distances, according to the bulk of the hydrocele. A drop or two of fluid escaping at the several needle orifices, a rag dipped in cold lotion was laid on the part, and the general result was more or less œdema of the scrotum, and in three days a total disappearance of the swelling. I soon ascertained that it was not by adhesive or any other mode of inflammation that the change was effected, but that the tunica vaginalis was left perfectly free and natural in its relation to the testis, and the scrotum of its proper weight, size, and figure, so that relapse was more to be apprehended. I was at first impressed with the idea that the tense condition of the tunic was essential to the curative effects of the puncture, and thus explained the difference of result from that obtained by complete evacuation and collapse of the sac. But this opinion I have had occasion to modify, finding that a freer discharge than could be obtained by the round needle, and the consequent partial collapse of the sac, was on the contrary more immediately and certainly productive of the infiltration of the cellular membrane, and consequent absorption of the fluid. I have therefore since employed a very fine trochar, smaller than any in common use, as the preferable instrument. My mode of proceeding is to put the scrotum on the stretch, in front of the testis, by embracing it with the extended thumb and fore-finger of the left hand; and placing the patient opposite a window on which the sun falls, or at all events a strong light, I command the view of the transparent bag so perfectly as to avoid veins and any point of accidental adhesion or thickening, which is always marked by a corresponding opacity. The punctures are made in a perpendicular direction and in quick succession, about equi-distant from each other, while the tunic is kept tense by graduated compression. Of several cases thus treated some have remained cured; others, apparently cured, have relapsed after a fortnight or three weeks, and one after three months; but I do not consider this a fair criterion of the value of the practice, because from the shape and size of the acupuncture needles chiefly used in these cases, the points on which I believe the success of the operation mainly depends were not accomplished. These are, a freer collapse of the sac by the removal of a sufficient portion of the fluid which the trochar puncture ensures, and the more complete diffusion of the remainder into the surrounding cremaster and cellular tunics, obtained by the multiplication of the punctures, while the tension of the sac is preserved. On the third day the fluid is absorbed, and the two sides of the scrotum are uniform: indeed, this is sometimes the case on the second day; but if the punctures are so small as not at once sensibly to reduce the bulk of the swelling, a single drop only exuding at each orifice, the reduction is generally much slower, or even fails altogether. *Med. Gazette, February* 11, 1837.

Mr. Travers imagined that he had anticipated Mr. Lewis in this mode of treating hydrocele; as he says he first conceived the idea of it in 1835, and mentioned it in his surgical course of lectures, previously to the publication of Mr. Lewis's first letter. In a subsequent communication in the Lancet of February 18th, Mr. Lewis informs us that he had performed the operation two years before the time mentioned by Mr. Travers. However, a letter in the Medical Gazette of the same date, from Mr. Keate, of Albemarle street, deprives both these gentlemen of the honour of having first employed this practice. The following extract from Mr. K.'s communication leaves no doubt on this head.

"While I do not mean to intimate any doubt of the same ideas having occurred to each of these gentlemen without any knowledge of the other's theory or practice, or of any previous operation of the kind, I trust it will not be offensive to either of them if I assure you that the plan and the practice have been known and acted on for very many years by myself, and I dare say by others. By one other person I know it was performed I dare say twenty years ago, namely, by a friend of mine, who for some years practised as a physician in this town, and is now living in retirement in the country. This gentleman performed the operation on *himself*, as he was nervous about the injection, and fancied, as he said, that if he could convert ascites into anasarca, absorption from the cellular structure might cure the malady; and in his own case it was perfectly successful. At his suggestion I tried it frequently, both at the hospital and in private practice; sometimes successfully, but more frequently the collection of fluid in the sac returned, and I generally found the patients impatient of the numerous punctures, and of the time required for the absorption. I remember talking to Sir Astley Cooper on the subject, and, as far as my recollection serves me, the plan appeared not to be new to him."

*Med. Gazette, February* 18, 1837.

---

*On Hydriodate of Potash in Secondary Syphilis.* By H. Bullock, m.r.c.s.

Mr. Bullock has reported the particulars of eleven cases of secondary syphilitic diseases, of a formidable character, relieved by hydriodate of potash, administered internally, in doses of eight grains three times daily, in camphor mixture. The symptoms were—destruction of the uvula and soft palate, or nodes, with nocturnal pains, on the tibia, ulna, frontal, and malar bones; and affection of the bones of the nose, or rupia and other tubercular eruptions. The period of cure was from one to two months. The patients were treated by Dr. Williams, at St. Thomas's Hospital. They were all adult males.

*Edinburgh Med. and Surg. Journal, Jan.* 2, 1837.

---

*Extirpation of an Inverted Uterus.* By W. Moss, Esq., Windsor.

This was a case of complete eversion following delivery, six years previously, and at the period of the operation the organ was entirely protruded from the vagina. The operation was attempted to be performed in the first place by ligature; Indian twist, whip-cord, gimp, and wire being successively used. Much pain and irritation, with nausea, &c., ensued from the use of the different ligatures; which, however, were persevered in, being occasionally tightened and relaxed according as they could be borne, until the twentieth day, when the remaining portion of the neck of the uterus was cut through with the knife; the organ having shrivelled to half its original size under the use of the ligatures, and, when removed, weighed seven ounces. There was a good deal of hemorrhage, and several arteries required to be secured. Although, during the progress of the cure, there was protrusion of the intestines per vaginam, and other distressing accidents, the woman was so well as to go to work about twenty days after the excision of the uterus. She still, however, continued subject to incontinence of urine, and required to wear a pessary, to prevent protrusion of the intestines.—Query: Would it not have been better to have employed excision in the first instance, and so have saved all the distress of the attempts with the ligature? *Lancet, Dec.* 3, 1836.

*Unusual State of the Contents of the Hernial Sac.* By HENRY TAYNTON, Esq.

IN a case of chronic inguinal hernia, which had been strangulated about thirty-six hours, and which could not be reduced, Mr. Taynton found, on exposing the sac in the operation, considerable difficulty "in pinching up a portion for the purpose of dividing it, on account of its great tenseness." "An opening being made, (continues Mr. T.,) about four ounces of serum gushed out. When the sac was laid open, a large mass of coagulable lymph presented itself, having the appearance of calf's-foot jelly, only of a darker colour, not adherent, and which totally concealed the protruded intestine. This jelly-like substance, which formed the bulk of the tumour, being removed in one large mass, a moderately-sized knuckle of intestine was observed, in a highly vascular state, with laminæ of lymph adhering to it, of the same colour as that contained in the sac. The sac itself was quite free from inflammation. The stricture being divided, the operation was completed in the usual manner." The man recovered.

*Med. Gazette, Jan.* 21, 1837.

*On Union of Fractures of the Patella.* By GEORGE GULLIVER, Assistant Surgeon to the Forces.

MR. GULLIVER gives two cases of bony union of fracture of the patella in the human subject. The first was the case of a sailor, who fell on his knee from the maintop of a brig: the second was that of a soldier, whose patella was fractured by a gun-shot. Mr. G. has also related a series of experiments on rabbits and dogs: he broke the patella in two ways: first, by blows, so as not to divide the aponeurosis which covers it, and in such cases the union was osseous; and secondly, by cutting the bone and aponeurosis in two with cutting forceps, and in these cases no bony union took place. His conclusions are, that, when the aponeurosis is completely divided, as in fractures of the patella from muscular violence, bony union is not to be expected; for in such cases it is impossible to keep the fragments in accurate contact; and that osseous union is simply the effect of immoveable coaptation of the fragments, the provision for which, in certain forms of fracture, (as from external violence, as falls on the knee and gun-shot and incised wounds,) is the integrity of the aponeurosis in front of the bone.

*Edinburgh Medical and Surgical Journal, Jan.* 1837.

*Case of Scalded Glottis.* By GEORGE FAYRER, Surgeon, of Barking.

THIS accident, which occurred in the usual manner, by the child (æt. seven,) attempting to drink from the spout of a tea-kettle, was treated according to the plan originally suggested by Mr. Wallace, of Dublin, and was speedily cured. Two grains of calomel were given every hour, and two minims of laudanum every two hours. On the third day the calomel showed its specific effect on the mouth, and the child speedily got well. *Lancet, Feb.* 11, 1837.

